# Knowledge and Attitudes toward Human Papillomavirus and Vaccination: A Survey among Nursing Students in Saudi Arabia

**DOI:** 10.3390/healthcare11121766

**Published:** 2023-06-15

**Authors:** Sally Mohammed Farghaly Abdelaliem, Abeer Mohammed Kuaia, Afnan Ahmed Hadadi, Alhanouf Khalid Alhujayri, Amal Awad Al Anazi, Areen Abdulelah Hajar, Ruba Shayaa AlShareda, Saleha Mohammed Amri

**Affiliations:** 1 Nursing Management and Education Department, College of Nursing, Princess Nourah bint Abdulrahman University, P.O. Box 84428, Riyadh 11671, Saudi Arabia; 2 Nursing Administration Department, Faculty of Nursing, Alexandria University, Alexandria 21544, Egypt; 3 College of Nursing, Princess Nourah bint Abdulrahman University, P.O. Box 84428, Riyadh 11671, Saudi Arabia; 441000558@pnu.edu.sa (A.M.K.); 441000254@pnu.edu.sa (A.A.H.); 441002383@pnu.edu.sa (A.K.A.); 441001041@pnu.edu.sa (A.A.A.A.); 441001787@pnu.edu.sa (A.A.H.); 441005337@pnu.edu.sa (R.S.A.); 441005333@pnu.edu.sa (S.M.A.)

**Keywords:** human papillomavirus, HPV vaccine, nursing students, knowledge, attitude

## Abstract

Introduction: One of the most prevalent conditions affecting the vaginal organs is the human papilloma virus (HPV). Human papillomavirus (HPV) knowledge and attitudes have been the subject of numerous studies in Saudi Arabia. However, there are only a few studies that have examined university students’ attitudes and knowledge of the human papillomavirus and the vaccine that is associated with it. Aim: To predict the level of knowledge and attitudes regarding HPV and its related vaccine among undergraduate nursing students. Methodology: This was descriptive cross-sectional research. After being selected from Princess Nourah bint Abdulrahman University’s College of Nursing, 307 nursing students agreed to take part and completed an online survey that was self-administered. Results: The majority of the participants (73.5%) had a low level of knowledge of HPV with a mean score of 2.77 ± 1.78. In addition, more than half of the participating nursing students (57%) had a moderate attitude toward HPV vaccination with a mean score of 51.18 ± 11.16. The study results also verified that there was a highly significant correlation between the nursing students’ demographics and their knowledge and attitudes toward HPV (*p* < 0.001). According to the SEM, nursing students’ knowledge regarding HPV accounted for 48% of the variance in students’ attitudes. Conclusion: Nursing students’ knowledge regarding HPV vaccination is an important predictor of students’ attitudes regarding HPV.

## 1. Introduction

Human papillomavirus (HPV) is one of the most common genital viruses. There are one hundred different types of HPV, which spread through skin-to-skin contact, including sexual contact and other types of contact with the genital region, such as touching the genitalia of an infected individual. HPV cannot be spread by coming into contact with objects such as a toilet seat [1]. In most situations, HPV infection is stopped by the immune system before it causes warts. When warts do develop, their appearance varies depending on the type of HPV infection [2]. Genital warts can appear as flat lesions, little cauliflower-shaped pimples, or stem-like protrusions [2]. Common warts typically develop on the hands and fingers and resemble rough, raised bumps. Foot warts: the heels or balls of the feet are also the typical locations for plantar warts, which are hard, granular growths. Flat warts are lesions with a flat top and a small amount of raised surface. They may appear anywhere [3]. The global incidence rate of genital HPV infections in men ranges from 3.5 to 45%, compared to 2 to 44% in women [4]. In Saudi Arabia, 31% of the general population, >92% of women with cervical cancer, and 80% of women with cytological abnormalities have had HPV infection. More than 80% of sexually active men and women will contract HPV in their lifetime, and there are more than 14 million new infections in the US each year [5]. In 2018, around 43 million people contracted HPV, many of whom were in their late teens and early twenties [3].

One of the most prevalent malignancies in women is cervical cancer, with developing nations accounting for the vast majority of new occurrences (more than 85%). Invasive cervical cancer and precancerous cervical intraepithelial neoplasia (CIN) are both caused by persistent human papillomavirus (HPV) infection. Between 75 and 80 percent of those who engage in sexual activity will contract HPV at some point in their lifetime. Thankfully, preventive HPV vaccinations have received widespread approval for the primary prevention of cervical cancer. Current research shows that these vaccines, such as the 2-valent Cervarix^®^ vaccine from GlaxoSmithKline, Wales; the 4-valent Gardasil^®^ vaccine from Merck Inc., Whitehouse Station; and the 9-valent Gardasil^®^9 vaccine from Merck Inc., Whitehouse Station, effectively prevent HPV infection and the emergence of cervical cancer [3,4,5].

Although the majority of HPV infections and precancerous lesions disappear on their own, some persist and develop into aggressive cervical cancer. Cervical cancer is the fourth most frequently diagnosed cancer in women globally and the fourth most common cause of cancer-related death. It is the 10th most prevalent malignancy in Saudi Arabian women between the ages of 15 and 44 [6,7]. Although the majority of HPV infections and precancerous lesions disappear on their own, some persist and develop into aggressive cervical cancer. Cervical cancer can be detected early by cervical screening tests such as the Pap test, but in Saudi Arabia, most cases are discovered in the advanced stages when treatment is challenging [8,9]. HPV is a significant cervical cancer risk factor. There are numerous HPV-related viruses or serotypes, and cervical cancer is highly associated with high-risk HPV serotypes. HPV 16 and HPV 18 are two often-found high-risk serotypes with carcinogenic qualities. Several studies also suggest that the Chlamydia bacteria may aid HPV in growing and persisting in the cervix, which may raise the risk of cervical cancer. Several preventive HPV vaccinations are being offered in Saudi Arabia [10,11,12,13]

Moreover, preventative HPV vaccinations for girls ages 11 to 26 have received approval from the Saudi Food and Drug Administration (SFDA) in 2010. Since then, HPV vaccinations have been added to the Saudi National Immunization Schedule’s list of required immunizations for females [14]. In addition to headaches, fatigue, and nausea, HPV vaccines can also result in discomfort, swelling, and redness where the shot was administered. The most frequent significant HPV vaccination adverse effects are lightheadedness and fainting. Although these are widespread misconceptions, there is no proof that HPV vaccines cause infertility or autoimmune illnesses [15]. According to Taebi et al. (2019) [16] researchers found that women, medical students, and nurses had little knowledge about the HPV vaccine.

### 1.1. Conceptual Framework

**Figure 1:**
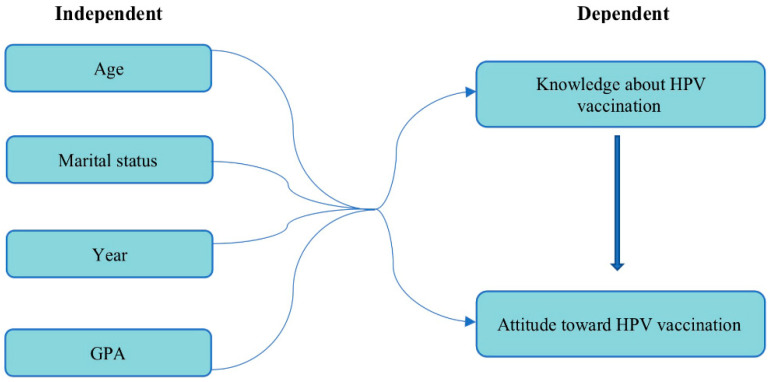
Conceptual framework on human papillomavirus (HPV) and HPV vaccinations among undergraduate nursing students at Princess Norah bint Abdulrahman University.

Figure 1 shows the variables in the study. The profile of the respondents include age, marital status, GPA, the academic year. The profiles include factors deemed to affect the students’ knowledge and how it will affect their attitudes regarding human papillomavirus and HPV vaccination among undergraduate nursing students.

#### 1.1.1. Statement of the Problem

A lack of knowledge about HPV and its related vaccine is very common worldwide, especially in Saudi Arabia. A study showed that only 34.5% of Saudi Arabian participants were aware of HPV, compared to 65.5% who had never heard about it. This is a considerably low level of awareness. The prevalence percentage is very high in Saudi Arabia. As studies have shown, 80% of women with cytological abnormalities, >92% of women with cervical cancer, and 31% of the general population are infected with HPV [17]. More than 80 percent of sexually active men and women will contract the infection in their lifetime, and there are more than 14 million new infections in the US each year [18]. In 2018, around 43 million people contracted HPV, many of whom were in their late teens and early twenties [13]. Since 2010, Saudi Arabia has had access to HPV vaccines (bivalent and quadrivalent), which have been introduced to the normal immunization schedules for girls in the Saudi National Immunization Schedule. Nonetheless, studies that evaluate the efficacy and safety of vaccines are scarce [14]. 

More than half of the individuals disagreed that HPV is a widespread sexually transmitted disease; furthermore, 63% of participants agreed that the HPV vaccine may prevent warts and cervical cancer, and 53% expressed interest in it. The vaccine was opposed by about 30% of the participants for religious reasons [14]. In terms of the research gap, there are several studies that have been completed worldwide in terms of knowledge and attitudes about HPV and its related vaccine [16]. Despite this, there have been no studies conducted on university students in Saudi Arabia, who are considered the target group because they are more likely to be infected with the papilloma virus, so it is extremely important to increase students’ awareness about HPV because they have a high risk of incidence because of their vulnerable age [10]. In addition, Lin et al., 2019 [10] revealed that there was a scarcity of published research regarding how to facilitate HPV vaccination (3). Their sample was women, the general population, aged above 15 years. In order to provide knowledge and increase protection from this virus and its problems, awareness can be enhanced in our community by advocating the HPV vaccine’s acceptance and use [14].

##### Knowledge about HPV and Its Vaccine 

The human papillomavirus (HPV) is one of the most prevalent STIs, which causes genital infections in about 5.5 million people each year [16]. Depending on each study’s questions, there were significant variations in the groups’ knowledge of HPV infection, although overall understanding and awareness of this STI were poor [16]. University students participated in a study that examined this topic, and the results revealed that the students’ awareness of and consideration for HPV infection were insufficient [19]. There were considerable gender variations in general knowledge of HPV, with more women (59.72%) than men (43.8%) reporting having heard of the disease [20]. Only 38.5% of the women had heard of HPV, according to Chen et al., 2021 [21], and 76% of the participants were considered to have limited knowledge (Taebi et al., 2019). More than half of the students stated that they were unaware of the HPV risk factors and route of transmission [19]. Another study revealed that 20.1% of students and hospital workers were unaware of the HPV transmission methods [16]. The study also discovered that students’ awareness of HPV as a cancer cause, prevention strategies, and diseases caused by HPV was insufficient [19]. Additionally, few people in the general population were aware of the connection between HPV and other malignancies [20]. 

The HPV vaccine has been shown to be effective in reducing genital warts and malignancies associated with HPV, particularly cervical cancer [6]. According to the study’s findings on the effectiveness of the HPV vaccination, just 7.2% of the participating university students reported they were aware of the vaccination, while 33.7% said they could be protected against HPV. Similarly, in Koç’s study, this incidence was 8.7%, and vaccination knowledge was inadequate. Just 10.9% of female university students in Pakistan reported knowing about the HPV [19]. According to the study of Cinar and Alatas (2019) [19], university students had a poor understanding of HPV infection, cervical cancer, and HPV vaccination. They also held limited beliefs and attitudes about these topics. Health education students were more familiar with the HPV vaccine and had more in-depth knowledge about it [19]. This study’s low vaccine adherence rate (less than one-third of respondents) can also be attributed to worry about the hazards associated with vaccinations. Monteiro et al., 2018 [22] observed that 33.1% of respondents from universities were worried about the HPV vaccine’s negative effects, which were also reported. Several negative remarks about the vaccine’s ingredients, its unknown side effects, and its role in the development of chronic diseases were found in a study on news websites conducted in Canada, revealing a lack of public knowledge of the vaccine’s benefits (Monteiro et al., 2018) [22].

##### Attitudes toward HPV and the HPV Vaccine

Darraj’s cross-country analysis (2022) [14] showed the majority of participants (50%) agreed that males should receive the HPV vaccine to protect themselves from HPV infection from a potential partner and its implications. Over 34% of participants believed that since women are already protected against HPV, men do not need to be protected as well. About 60% (n = 569) of participants felt that HPV vaccination is necessary to protect females from developing vaginal and anal warts. In addition, 50% of respondents said vaccination was unnecessary since females are too rarely affected by HPV-related malignancies. About 33% of participants thought there were other approaches to treating warts in women than vaccination, and 48% of participants felt that vaccination of females against HPV was necessary to prevent males from contracting the virus. Only 21% of participants disagreed that vaccination against HPV is too late if a male adolescent is already sexually active. A third of participants were afraid that the vaccination might promote riskier or earlier sexual behavior; 38% thought the vaccine was too new; and 45% were worried about vaccine safety. A total of 29% of people rejected HPV vaccination for moral or religious reasons, and roughly 47% were not aware that vaccinations were available for both males and females. About 53% of men were interested in the HPV vaccine, while 36% were worried about the price [14].

Another study showed a positive attitude toward HPV was linked to screening intentions. Informed nurses and midwives might have favorable opinions of HPV vaccination. The recommendation of the vaccine by medical experts will increase its acceptance, according to a comprehensive review of the acceptability of HPV vaccines in sub-Saharan Africa. As a result, effective advice from medical professionals may impact HPV vaccination behavior. The barriers to HPV vaccination according to a group of people consisted of 12 items, such as they thought getting the HPV vaccine was a waste of time, there were no vaccination sites available, and there was not much information available about the HPV vaccine. In addition, they did not know what the HPV vaccine was for, there were no health education programs to promote the HPV vaccination, the vaccine was not necessary. They also said they did not have the money to get the vaccine, they were afraid of the vaccine’s side effects, and there was a risk of contracting the disease [1,23]. By asking participants to rate eight pertinent statements using a Likert scale, attitudes about the HPV vaccine and immunization in general were ascertained. 

The HPV vaccine and vaccination in general were viewed favorably by the students. The HPV vaccine was considered to be effective and safe, according to more than 70% of the participants. About 21% of the participants tended to agree that vaccination is not necessary for healthy people. Of the 226 responders, 143 (63.3%) mentioned that there was a possibility of HPV infection even at a young age of 9 to 14 years old. Of the 226 students, 204 (90.3%) emphasized the need for the HPV vaccine to be included in the NIP in the future [24]. The majority of the female nurses and women were unaware that vaccination against HPV could prevent cervical cancer. In a study completed in China about gender differences in attitudes toward the HPV vaccine, female college students who had heard about the HPV vaccine had a higher willingness to receive it, while there was no effect on males. This may be because the term (cervical cancer vaccine) is frequently bound with HPV vaccine, leading one to believe that it is not relevant to men. More precisely, the higher level of awareness about the HPV vaccine that female college students possessed was a significant determinant of their HPV vaccination [21]. In Spain, the two-dose vaccination schedule had an average coverage percentage of 79.0% for girls in 2019 [25]. 

Low HPV vaccination rates were observed in several studies. It was discovered that just 13.3% of undergraduate students in Hong Kong research had received an HPV vaccination. Only a small number of nurses in Turkey reportedly received the vaccination. The results of this research strongly imply that the issue of low vaccination rates against HPV infection is a global problem. However, health education among medical professionals was found to positively affect the intention to get the vaccine and perhaps suggest it to others in the future in studies conducted in different populations such as Istanbul, Turkey, and South India [24,25,26]. 

##### Factors Affecting HPV and the HPV Vaccine

This study has more recently focused on factors affecting HPV and HPV vaccines, with genital warts seen as a common side effect of some sexually transmitted HPVs, while other side effects can lead to a variety of malignancies, including those of the cervix, vagina, vulva, penis, anus, and oropharynx [13]. A specific cellular form of cancer known as an HPV-associated cancer is one that is discovered in a body region where HPV is frequently present. According to estimates, the virus is to blame for 79% of malignancies linked to HPV [13]. In Mississippi (MS), the incidence of HPV-associated malignancies is estimated to be 14.3 per 100,000 people, higher than the 11.7 per 100,000 people nationwide, according to data from cancer registries. Moreover, vaccine recipient’s characteristics, such as younger age, Caucasian race, higher education level, higher household income, and health insurance coverage are factors associated with a greater likelihood of vaccine series completion, according to studies reporting HPV vaccine completion rates ranging from 23% to 60%. Regarding the Medicaid population, nothing is known about the variables that affect vaccine series completion. According to estimates, 60% of Mississippians under the age of 19 were registered in Medicaid fee-for-service (FFS), the Children’s Health Insurance Program (CHIP), or Medicaid’s senior care programs as of July 2017. Given the high prevalence of cancer linked to HPV [18,27,28], the current study looked at the HPV vaccine series completion rate and variables associated with completion among MS Medicaid beneficiaries aged 9 to 26 years, as well as the sizeable Medicaid-served population in the state. Other studies have shown that continual HPV infection can result in conditions including genital warts, precancerous lesions, and certain malignancies. In nations where the HPV vaccination is licensed, the literature has examined the factors that influence adolescents’ and their parents’ understanding about HPV and acceptance of the vaccine. In a recent comprehensive review of the literature, which looked at seventy non-interventional studies carried out in 16 European nations, the primary variables linked to HPV knowledge were female gender, higher education, and higher income group [29].

One study by Namakula (2018) reported that cervical cancer is the main consequence of human papillomavirus (HPV) infections. Cervical cancer is the fourth most prevalent cancer in women, affecting 500,000 of them annually and killing an estimated 266,000 people. With an age-standardized incidence rate of 47.5 people per 100,000, Uganda has one of the highest incidences of cervical cancer worldwide. This study evaluated the extent to which female teenagers in the Lira area of Uganda had received the HPV vaccine as well as the contributing factors. A survey of 460 female teenagers was conducted using a mixed-methods technique. The information was gathered through a questionnaire that an interviewer administered. Ten in-depth interviews were conducted and five important sources were spoken with. Uptake was outlined as receiving all three required doses of the vaccination. The respondents’ average age was 13.97 (SD: 1.24) years. There was a 17.61% uptake (81/460). The Lira district had a poor rate of HPV vaccination uptake. Ensuring a steady supply of vaccinations at the vaccination sites, health education aimed at fostering a good attitude about the vaccine, sensitizing teenagers about the vaccine, and undertaking community outreach should be the main priorities of efforts to increase the HPV vaccine uptake [30].

#### 1.1.2. Aim

The aim of the study is to predict the level of knowledge and attitudes regarding HPV and its related vaccine among undergraduate nursing students.

##### Research Questions

The researchers aim to assess the level of knowledge and attitudes toward human papillomavirus and its related vaccine among undergraduate nursing students at Princess Nourah bint Abdulrhman University. Specifically, it will answer the following:

1. What is the socio-demographic profile of undergraduate nursing students at Princess Norah bint Abdulrahman University in terms of: age, marital status, GPA, and academic year?
2. What is the level of knowledge of HPV among nursing students?
3. What is the attitude toward the HPV vaccine among nursing students?
4. What are the factors affecting nursing students’ knowledge and attitude regarding HPV?
5. What is the relationship between the nursing students’ demographics and their knowledge and attitude?

#### 1.1.3. Hypothesis

Alternative hypothesis: There is a correlation between demographics and knowledge and attitudes toward HPV among undergraduate students at Princess Nourah bint Abdulrahman University.

Null hypothesis: There is no correlation between demographics and knowledge and attitudes toward HPV among students at Princess Nourah bint Abdulrahman University.

## 2. Materials and Methods

Research Design: A descriptive cross-sectional quantitative research design was used.

Research Setting: The study setting was the College of Nursing—Princess Nourah bint Abdulrahman University.

Population: A total of 1014 students from Princess Nourah bint Abulrahman University made up the study population. Participants were undergraduate nursing students.

Inclusion criteria were as follows: undergraduate nursing students (enrolled in the nursing program from the 1st year to the 4th year) at Princess Nourah bint Abdulrahman University.

Exclusion criteria included students enrolled in internships, midwifery students, diploma and masters’ students, anyone with a life-threatening allergic reaction to the HPV vaccine or any of its components, anyone with a history of severe allergies or a yeast allergy, and patients with moderate or serious disease, those who are not willing to participate in the study.

Sampling: The sample included only undergraduates nursing students at the College of Nursing—Princess Nourah bint Abdulrahman University (n = 307) which represented as follow; 1st year represented 51% (n = 156), 2nd year represented 25% (n = 77), 3rd year represented 13% (n = 39), and 4th year represented 11% (n = 35) from participants with 100% response rate. Based on the Epi Info program calculation, with a margin of error of 5%, the confidence level is 95%, sampling attrition 10%, sample size was calculated. So, from the total population of 1014 nursing students, a sample size of 307 undergraduate nursing students was included in the study (Tables S1 and S2 (Supplementary Materials)). The researchers used the raosoft website to calculate the sample size: http://www.raosoft.com/samplesize.html (accessed on 16 March 2023).

Sampling Technique: The type of sampling was non-probability convenience.

### 2.1. Research Instruments

The validated Knowledge and Attitude toward HPV questionnaire (KAHPVQ) which was developed by White et al., 2016 and translated into Arabic by Darraj et al., (2022) [14] was utilized. Permission to use the questionnaire was given. A brief introduction preceded 35 closed-ended questions with predefined options. There were five sections in the survey. The purpose of this study, scope, and estimated time of completion were described, along with details on informed consent. In the first section, participants’ demographic and medical data were collected, including age, marital status, family history, year, GPA, and uptake of the HPV vaccine (6 items). The second section asked about participants’ knowledge about HPV in general (9 items) on a 3-point Likert scale (0 = don’t know to 2 = true). On a 5-point Likert scale, participants were asked about their awareness of HPV in females in the third section (1 = strongly disagree to 5 = strongly agree). In addition, the 4th section focused on items about participants’ attitudes toward the HPV vaccination (7 items) on a 5-point Likert scale (1 = strongly disagree to 5 = strongly agree). Finally, the 5th section included items about participants’ attitudes toward the HPV vaccine in general (8 items) on a 5-point Likert scale (1 = strongly disagree to 5 = strongly agree). The average score was calculated for each section, and the total scale; scores ranging from 2.51 to 3.50 indicated a moderate level of knowledge and attitude, scores lower than 2.51 indicated a lower level of knowledge and attitude, and scores higher than 3.50 indicated a higher level of knowledge and attitude regarding HPV.

### 2.2. Validity and Reliability

The design of the questionnaire was based on an analysis of pertinent studies [11,12,13,14]. They assessed the usefulness, specificity, and thoroughness of the questionnaire items for validation. The participants’ native language was colloquial Arabic, so the Arabic validated version of the questionnaire was utilized. The generated questionnaire’s content and capacity to capture the data required to address the study’s research topic were already evaluated. Additionally, a pilot study was conducted with 30 people from the target population to test the clarity of the questions and the length of the interview. Several questions were revised in response to their input. The results of the pilot study were not included in the final results. The internal consistency reliability of the questionnaire was determined to be adequate (0.870).

### 2.3. Data Collection Procedure

Data were gathered from April to May 2023 with an IRB approval from Princess Nourah bint Abdulrahman University and administrative clearance from the dean of student affairs. The survey was developed online on Google forms and distributed among the study sample through emails via the student affairs offices of the College of Nursing and Deanship of Scientific Research, and WhatsApp groups. The questionnaire took about 15 min to be completed by the participants.

### 2.4. Statistical Analysis

IBM SPSS Statistics version 26 and IBM SPSS AMOS version 23 were used for all statistical analyses. Descriptive statistics (frequency, means, standard deviations, and percentages) were used to quantify demographic characteristics, whereas inferential statistics (Student’s *t*-test and analysis of variance (ANOVA) were used to compare study variables between groups based on socio-demographic characteristics. The correlation coefficient was utilized to examine the relationship between the variables in the study. A multiple regression analysis was performed to predict the knowledge and attitude score in response to demographics. The variables included in the multiple regression models as independent variables were those that were statistically significant (*p*-value 0.05) in the correlational analysis, with a correlation coefficient of 100. The structural equation modeling method was also used to analyze the relationship between the research variables. Structural equation modeling is a powerful data analysis technique for investigating complex interactions between multiple analytical variables. It may simultaneously test several mediating and moderating interactions, estimate latent variables using related measures, and deal with practical issues such as non-normality and missing data. Demographics were deemed an independent variable by the authors, while students’ knowledge and attitudes concerning HPV were considered dependent factors. The model’s assumptions were shown to be true. All statistical analyses were performed using IBM SPSS Statistics version 23 and IBM SPSS AMOS version 23. All *p*-values shown are two-tailed, and the 0.05 threshold was used for statistical significance.

### 2.5. Ethical Considerations

IRB approval was obtained from Princess Nourah bint Abdulrahman University with IRB Number (HAP-01-R-059/23-0397). Data confidentiality and privacy were sustained and guaranteed. The participants’ written agreement to participate in the research was obtained before the collection of safeguarded data. The study subjects were ensured the right to withdraw from the study.

## 3. Results

Table 1 shows that the highest percentage (77.9%) of the study sample was in the age group aged 18–20 years old. In relation to marital status, the highest percentage of the nursing students (97.1%) were single. In terms of GPA, 60.6% of the nursing students’ grades had a GPA that ranged from 3.51 to 4.50. In relation to the academic year of the nursing students, the results showed that more than half of the study sample were enrolled in the first year of the nursing program, followed by 25.1% of the nursing students who enrolled in the second year of the nursing program. Regarding the study sample’s family history of cervical cancer, the findings indicate that 81.4% had no family history of cervical cancer. In terms of the study sample’s ability to receive the HPV vaccine, it was found that 89.3% did not receive the HPV vaccine.

According to Table 2, the findings revealed that 50 % of the study sample answered correctly regarding Item 3 “the incidence of HPV in women is highest among women in their 30s’’, and Item 8 “the HPV vaccine is available for both females and males” with a mean score of 0.52 ± 0.50 and 0.52 ± 0.50, respectively. Moreover, 77.5% of the nursing students had a low level of knowledge regarding the HPV vaccine in general, with a mean score of 2.77 ± 1.78. In addition, the highest percentage (50.1%) of the study sample answered correctly regarding Item 3 “Genital and anal warts can cause serious physical, emotional, and financial consequences for females’’ with a mean score of 0.52 ± 0.50; however, the rest of the items regarding the HPV vaccine in general and in females were answered incorrectly. In addition, 66.8% of the nursing students had a low level of knowledge regarding the HPV vaccine in females, with a mean score of 1.82 ± 1.73. Furthermore, the study results indicated that nursing students had a low level of knowledge regarding the HPV (with a mean score of 4.59 ± 3.06.

According to Table 3, the findings revealed that the highest percentage of nursing students had a moderate attitude level toward HPV vaccination (57%) with a mean score of 51.18 ± 11.16. In addition, more than half of the nursing students had a moderate attitude level toward female vaccination (55.7%) with a mean score of 23.86 ± 5.52. The highest mean score was related to Item 3 “the important to vaccinate female against HPV to prevent them from getting genital and anal warts” with a mean score of 4.11 ± 0.92, and the nursing students had a high attitude level of 69.4% towards this item. Furthermore, the lowest mean score was related to Item 7 “it’s too late to vaccinate against HPV if an adolescent female is already sexually active” with a mean score of 2.83 ± 1.35, and the nursing students had a low attitude level of 38.4% towards this item.

In addition, Table 3 showed that the highest percentage of nursing students’ total attitude toward the vaccine was moderate (51.5%), with a mean score of 27.33 ± 6.71. As the highest mean score was related to Item 6 “I am interested in the HPV vaccine for female” with a mean score of 3.92 ± 1.12, and 65.5% of nursing students had a high attitude level towards this item. The lowest mean score was related to the Item 4 “opposed to HPV vaccination for moral or religious reasons” with a mean score of 2.57 ± 1.45, as 51.8% of nursing students had a low attitude level towards this item.

Regarding the correlation matrix in Table 4, there was a highly significant positive correlation between the nursing students’ overall knowledge and overall attitudes toward HPV vaccination in females, and attitudes toward the HPV vaccine in general (r = 0.557, *p* < 0.001, r = 0.579, *p* < 0.001, and r = 0.483, *p* < 0.001, respectively). There was also a highly significant positive correlation between overall knowledge and the nursing students’ marital status, GPA, family history of cervical cancer, and receiving the HPV vaccine (r = 0.150, *p* = 0.009, r = 0.277, *p* < 0.001, r = 0.388, *p* < 0.001, and r = 0.215, *p* < 0.001, respectively). However, a highly significant negative correlation was found between overall knowledge and nursing students’ age and academic year (r = −0.154, *p* = 0.007, and r = −0.133, *p* = 0.019, respectively). Furthermore, there was a highly significant positive correlation between the nursing students’ overall attitudes and knowledge of HPV vaccination in females, and knowledge of the HPV vaccine in general (r = 0.425, *p* < 0.001, and r = 0.582, *p* < 0.001, respectively). There was also a highly significant positive correlation between overall attitude and the nursing students’ marital status, GPA, family history of cervical cancer, and receiving the HPV vaccine (r = 0.196, *p* = 0.001, r = 0.244, *p* < 0.001, r = 0.417, *p* < 0.001, and r = 0.195, *p* = 0.001, respectively). However, a highly significant negative correlation was found between overall attitude and nursing students’ age and academic year (r = −0.301, *p* < 0.001, and r = −0.361, *p* < 0.001, respectively).

To validate the relationship between the demographic data, their knowledge, and attitudes toward HPV vaccination, a regression analysis was performed, when the demographic data are the independent variables and knowledge and attitudes are the dependent variables Table 5. Because there was a significant relationship between demographic factors (marital status, GPA, academic year, family history of cervical cancer, receive HPV vaccine) and knowledge and attitudes, it was put into the regression equation. According to the regression analysis, a direct significant relationship was between knowledge of HPV and GPA (B = 6.318, Beta = 0.196, t = 3.708, *p* ≤ 0.001, LL = 2.965, UL = 9.671). The second factor affecting knowledge is a family history of cervical cancer with a direct relationship (B = 12.244, Beta = 0.309, t = 5.596, *p* ≤ 0.001, LL = 7.938, UL = 16.550), (R2 = 0.217, F = 13.860 *, *p* < 0.001 *). In addition, for attitude toward HPV, the most important demographic factor affecting attitude was marital status (B = 14.814, Beta = 0.135, t = 2.932, *p* = 0.004, LL = 4.872, UL = 24.756). Additionally, there was a direct significant relationship between attitude towards HPV and GPA (B = 2.515, Beta = 0.091, t = 2.046, *p* = 0.042, LL = 0.096, UL = 4.934). However, for the academic year it was a significant negative relationship with attitude regarding HPV (B = -5.235, Beta = −0.291, t = 4.387, *p* ≤ 0.001, LL = −7.583, UL = −2.887). The last factor of the demographic data affecting attitude is family history of cervical cancer (B = 5.349, Beta = 0.015, t = 3.297, *p* = 0.001, LL = 2.157, UL = 8.542). Another important factor is knowledge which had a significant relationship with attitude regarding HPV (B = 0.371, Beta = 0.436, t = 9.113, *p* ≤ 0.001, LL = 0.291, UL = 0.451), (R2 = 0.465, F = 37.146 *, *p* < 0.001 *).

**Figure 2:**
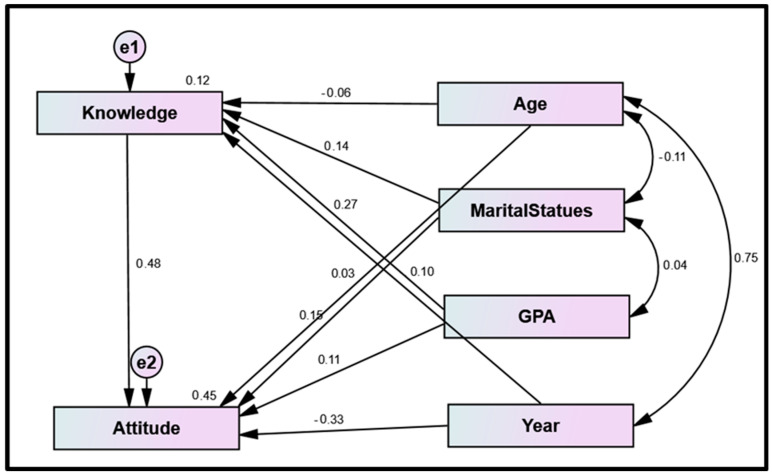
Structural Equation Modeling: Path Analysis for direct and indirect effect of the demographics on nursing students’ knowledge and attitudes (n = 307).

Figure 2 shows that academic year was negatively predicted in relation to attitude, whereas knowledge, age, marital status, and GPA exhibited a significant direct effect on nursing students’ attitudes toward the HPV vaccination. Similarly, GPA, marital status, and academic year exhibited a significant direct effect on nursing students’ knowledge regarding HPV vaccination. In addition, Figure 2 shows the path analysis model drawn with SPSS-AMOS clarifying the standardized regression weights of the structured equation modeling (Model X2 = 31.932; *p* < 0.001; Model fit parameters (CFI = 0.999; GFI = 0.998; RMSEA = 0.023). Nursing students’ knowledge accounted for the prediction of 48% of the variance in nursing students’ attitudes. All observed variables in the examined model were highly significant at *p*-value < 0.001 with strong estimates for the study variables (see Table 5 and Figure 2).

Model fit parameters CFI; IFI; RMSEA (0.999; 0.998; 0.023).

CFI = Comparative fit index; IFI = incremental fit index; RMSEA = Root Mean Square Error of Approximation.

Model χ; significance 31.932 *(<0.001 *)

## 4. Discussion

Human papillomavirus (HPV) is one of the most common STIs, infecting around 5.5 million people each year [16]. The current study results revealed that the highest percentage of the single nursing students were within the age group ranging from 18 to 20 years old in the first year of the nursing program, had a GPA ranged from 3.51 to 4.50, had no family history of cervical cancer, and most of them did not receive the HPV vaccine. These study results were supported by Concetta Paola Pelullo et al., (2019) [31] who found that the students’ average age ranged from 18 to 46 years old, 65.5% of them were female and practically all of them were single. Additionally, the current study results were supported by Sevgül Dönmez et al., (2019) [27] who revealed that 2.8% of the students had received a vaccination. Moreover, a study conducted in Turkey concluded that 23.9% of nursing students had received the HPV vaccine [31].

The current study results revealed that nursing students had a low level of knowledge regarding the HPV vaccine. This result may be due to the fact that nursing students have no experience regarding the human papilloma virus, as they did not study it in the academic curricula, there was a lack of educational intervention and awareness campaigns to augment the HPV immunization program, the family was not aware of the importance of vaccination against the human papilloma virus, and there was no awareness at the community level regarding the human papilloma virus and the importance of vaccinations. This study results were supported by White et al., 2016 and Gang Chen et al., (2022) [9] who revealed that college students had low knowledge of HPV and the HPV vaccine with a mean score of 22.73 ± 9.01. In addition, the current study was supported by Ilgun O Cinar et al., (2019) [19] who found that 33.7% of participating college students reported receiving the HPV vaccine, and only 7.2% of students reported knowing about the vaccine. Additionally, the current study results were supported by Mahboubeh Taebi (2019) [16], who conducted a study among women, medical students, and nurses and showed that little was known about HPV vaccination.

More than half of the nursing students had a moderate attitude level toward female immunization, according to the findings of the current study. The outcome may be related to the fact that females may feel that they need more protection and they may perceive that the HPV can be transmitted to the baby if the woman becomes pregnant. These results may be supported by those of He and He (2018) [32], who demonstrated that, despite the fact that the majority of women in Western China lack a fundamental understanding about HPV, at least half of them were willing to receive the HPV vaccine. Moreover, the majority of nursing students had a generally positive attitude; their study sample had pupils who showed a high level of acceptance toward the vaccine (95.8% acceptance rate). Furthermore, Sallam et al., (2021) [33], revealed that 75% of people would be open to receiving the HPV vaccine for free, compared to 16.0% who would pay for it.

The current study results revealed that there was a significant correlation between the nursing students’ age and their knowledge and attitude regarding HPV. This result may be due to the growth and development stage of the female nursing students in the age group of adolescence and adulthood as they became interested in women’s health awareness. The current study findings are contradicted by Shakurnia et al.’s (2022) [34] study results; there was no significant relationship shown between participants’ age and their awareness of or attitudes against HPV [34]. In contrast to the current study, Patel et al.’s (2016) [23] study found that adolescents had a limited awareness of HPV and the HPV vaccine. The findings of this study also conflicted with those of Shetty et al. (2019) [35], who found that participants under the age of 22 were less likely to accept the vaccine than participants over the age of 22. Furthermore, the current study results revealed that was a significant positive correlation between the nursing student’s material status and their knowledge and attitude regarding HPV. Because the women were at the stage when marriage was possible, they may be interested in knowing more about sexual diseases and how to protect themself from the diseases. According to Bodson et al.’s (2017) [34], study, which is supportive of the current study, individuals were considerably less likely to have heard of HPV when controlled for marital status. The findings of Ebu et al. (2021) [36], provide support. In addition, the current study results revealed that was a significant correlation between the nursing students’ GPA and their knowledge and attitude regarding HPV. This could be due to students with high GPAs tending to search more about the diseases that they studied in university courses, to obtain more knowledge and a better GPA, and tend to take the HPV vaccine when they know its importance. These results were supported by Shakurnia et al., (2022) [37], who revealed that higher age, a better GPA, and being in a group of medical students were significant odds ratio predictors of strong awareness/knowledge. Farsi et al., (2021) [38], also found that students who had a high GPA of 93.5% knew something about the human papilloma virus and the vaccine that is related to it. In contrast, the current study results were inconsistent with Shetty et al. (2019) [35], who validated that the participants’ grade point average (GPA) and HPV awareness and attitudes did not significantly correlate.

The current study results revealed that was a significant correlation between the nursing students’ academic year and their knowledge and attitude regarding HPV. This could be due to some students in the lowest academic year having more knowledge than those in the highest years; they may attend workshops and receive more health education at younger ages regarding sexual diseases. According to Baryamoglu Tepe and Ozcorekci (2020) [6], the university group provided much more accurate answers. In addition, Kellogg et al. (2019) [39], found that the greatest degree of education in the household had a substantial effect on immunization rates. These findings, however, are at odds with a recent study by Ebu et al. (2021) [36] who found a substantial correlation with complete educational level and support for HPV vaccination. The current study results revealed that was a significant correlation between the nursing student’s family history of cervical cancer and their knowledge and attitude regarding HPV. This may be due to students who have a family history of cervical cancer receiving education from health education team regarding HPV and the benefits of receiving its vaccine. This could be supported by Yörük et al. (2016) [40], who found that students with a family history of cancer were more inclined to think about being vaccinated. Ebu et al. (2021) [36] also found that having the HPV vaccine reduced the perceived risk of cervical cancer.

The current study results revealed that there was a significant correlation between the nursing students receiving the HPV vaccine and their knowledge and attitudes regarding HPV. When students have knowledge about HPV and how to protect themselves from it, this may influence the intent to receive the vaccine and the rate at which it is obtained. The findings of the research demonstrated by Oh et al. (2021) [41] was supportive of the current study findings, which demonstrate a higher perceived value of vaccination with more understanding about HPV and the HPV vaccine. In contrast to Bal-Ylmaz and Koniak-Griffin’s (2017) [8] study, a different study has shown that 98.1% of participants had high levels of knowledge of the risk factors and mechanisms of transmission for HPV. However, this understanding did not lead to the adoption of healthy habits, such as receiving an HPV vaccination. According to the supporting data of the study of Berenson et al. (2021) [15], 76% of nursing students reported starting the HPV vaccine, compared to 49% of medical students. The researchers Singh and Baliga (2021) [42] also found that females were more likely to receive vaccinations in the future.

The findings of the current study showed a substantial relationship between nursing students’ knowledge about HPV and their GPA. This result can be attributed to the students’ desire to learn more about the topics covered in their educational courses. This study is supported by the findings of Shakurnia et al., (2022) [34], which indicated that medical, nursing, and midwifery students with higher GPAs had much more awareness of and knowledge of HPV than those with lower educational accomplishment. In addition, the results of the current study indicated an important relationship between family history of cervical cancer and HPV knowledge. This might be probable because the most common cancer linked to HPV is cervical cancer. Furthermore, the results of this study show a significant relationship between married students and attitudes related to HPV. It could be because women become more concerned about women’s health awareness after they get married. Married students were less likely to have received the HPV vaccine than were single students, according to a study sponsored by Hollins et al. (2021) [43], who found that there was a substantial association between HPV vaccination and marital status. D’Errico et al. (2021) [44], reported that married and divorced students in a public university have higher positive attitudes concerning HPV than those who were never married, which is consistent with our findings. In addition, another result of our study showed that there was a significant relationship between attitudes towards HPV and GPA; this may be due to their knowledge about how important it is to be vaccinated against HPV currently and may be due to their study of health-related subjects.

Furthermore, the current study results were in contradiction with those of Shakurnia et al., (2022) [34], who found no significant correlation between students’ attitudes on HPV and their GPA. In this study’s results, there was a significant negative relationship between attitudes towards HPV and the academic year. This result may be due to students in their first years of nursing being more interested in knowing and learning more about health-related subjects. However, this result was contradicted by Shakurnia et al. (2022) [34], who said that midwifery students in higher academic years had a more positive attitude towards HPV and the HPV vaccine. The current study results revealed that there was a significant relationship between attitudes towards HPV and a family history of cervical cancer. Students who had a family history of cervical cancer had a higher positive attitude toward HPV and the HPV vaccine than students with no family history. Furthermore, this result may be because they have better knowledge and awareness about HPV because of knowing members of their families with cervical cancer. This result was supported by D’Errico et al. (2021) [44], who found that students who had a family history of cervical cancer had a more positive attitude toward HPV and the HPV vaccination. The current study showed that there was a relationship between attitude towards HPV and knowledge of HPV. This result may be because of the importance of being vaccinated against HPV. This study is consistent with the findings of the study by D’Errico et al. (2021) [44], who said that participants who had higher HPV knowledge were more likely to have a more positive attitude toward HPV and the HPV vaccination.

Strength and limitation: The present study’s findings have greatly added to previous HPV studies. However, the study’s weaknesses should be addressed. Because the sample size was limited to a specific group of people and the participants were recruited from a specific group in a specific context for convenience sampling, the results’ generalizability is limited. One of the study constraints was that the College of Nursing at PNU had a female-dominated educational environment. Furthermore, the current findings were prone to response bias and subjectivity because they were based on self-reported data. Furthermore, this study merely found correlations between study variables; no causal relationship was found. Longitudinal, experimental, and multi-site research may be useful in the future.

## 5. Conclusions

According to the current study’s findings, there is a considerable lack of information and attitude concerning HPV and its vaccine. Furthermore, nursing students have limited understanding about HPV and its vaccine. Today’s nursing students are the future nurses who will play a vital role in spreading HPV awareness and preventing cervical cancer. In order for students to appropriately inform the public as future healthcare practitioners, they must have a basic understanding of the HPV virus and its vaccine. The study’s findings highlight the importance of educational activities to increase students’ understanding and awareness of HPV in relation to future professions as health care workers.

Recommendations: Because of their critical role in public health education, nurses’ awareness and understanding of HPV will be among the primary determinants of health-care utilization. To raise awareness of HPV and its vaccine, a variety of techniques, including educational events and curriculum adjustments, should be implemented. Furthermore, clinician recommendation is an important factor influencing the parents’ decision to vaccinate their children, and school-based vaccination programs increase HPV vaccination rates by increasing vaccine access and the ability to reach a large population, engaging both the family and the clinician, and including the HPV vaccine in the Saudi national vaccination schedule.

### Implication of the Study

Practice: the current study findings will help in providing health awareness for the age group ranged from 18 to 26 to enhance their knowledge and understanding regarding the importance of receiving the HPV vaccine and its prevention.

Education: the results will help to build and implement different educational programs and training courses about the human papillomavirus and its related vaccine for the specified age group of nursing students.

Research: the study results will help the researchers to conduct more scientific research and studies with focus on increasing the number of the sample among female students at the university from various colleges, not just health colleges and among different sectors within the society.

Significance of the study in relation to different domains:

Nursing Education: This study will highlight the importance of implementing national educational programs about HPV and vaccine campaigns directed toward the public. This action would increase public knowledge of HPV and consequently enhance public attitudes toward the HPV vaccine.

Nursing Practice: This study will help enhance the knowledge and understanding of nurses to educate their patients in different community settings including hospitals, clinics, schools, and associations about the importance of taking the HPV vaccine and its preventive effects.

Community: The study will promote the HPV vaccine acceptance and use; awareness can be raised in our community to assure better knowledge and achieve higher protection from this virus and its complications by using advertising materials such as performing awareness campaigns in hospitals, malls, and schools.

Research: This study may contribute as the base line for further researchers regarding increasing awareness of the virus by applying nursing education sessions for students.

## Figures and Tables

**Table 1:** Distribution of the studied sample according to demographic data (n = 307).

	No	%
**Age**		
18–20	239	77.9
21–23	62	20.2
24–26	6	2.0
**Marital status**		
Single	298	97.1
Married	9	2.9
**GPA**		
1.50–2.50	10	3.3
2.51–3.50	75	24.4
3.51–4.50	186	60.6
More than 4.50	36	11.7
**Year**		
1st year	156	50.8
2nd year	77	25.1
3rd year	39	12.7
4th year	35	11.4
**Family history of cervical cancer**		
No	0	81.4
I do not know.	39	12.7
Yes	18	5.9
**Did you receive HPV vaccine?**		
No	274	89.3
Yes	33	10.7

**Table 2:** Mean and standard deviation for nursing students’ knowledge of HPV. (n = 307).

	Knowledge of HPV	Mean ± SD	Incorrect Answer		Correct Answer	
			No	%	No	%
1	HPV is a relatively uncommon sexually transmitted infection	0.19 ± 0.39	249	81.1	58	18.9
2	Almost all cervical cancers are caused by HPV infection	0.43 ± 0.50	174	56.7	133	43.3
3	The incidence of HPV in women is highest among women in their 30s	0.52 ± 0.50	147	47.9	160	52.1
4	Most people with genital HPV infections are symptomatic	0.22 ± 0.42	239	77.9	68	22.2
5	Genital warts are caused by the same HPV types that cause cervical cancer	0.11 ± 0.31	273	88.9	34	11.1
6	Sexually active adolescents should be tested for HPV before starting HPV vaccination	0.13 ± 0.34	267	87.0	40	13.0
7	HPV vaccine is not licensed for females older than 26 years of age	0.26 ± 0.44	228	74.3	79	25.7
8	HPV vaccine is available for both females and males	0.52 ± 0.50	146	47.6	161	52.4
9	Women and men who have been diagnosed with HPV should not be given HPV vaccine	0.38 ± 0.49	189	61.6	118	38.4
	Total of Knowledge of HPV	2.77 ± 1.78				
	Low	238 (77.5%)				
	Moderate	66 (21.5%)				
	High	3 (1.0%)				
1	Female are at risk for HPV infection	0.45 ± 0.50	170	55.4	137	44.6
2	HPV infection is common among female	0.37 ± 0.48	192	62.5	115	37.5
3	Genital and anal warts can cause serious physical, emotional and financial consequences for female	0.52 ± 0.50	148	48.2	159	51.8
4	Nearly all sexually active females have already been infected with HPV by age 26	0.18 ± 0.39	251	81.8	56	18.2
5	HPV infections may contribute to anal, vulval, lining of the vagina, cervical and rectal cancers in females	0.30 ± 0.46	215	70.0	92	30.0
	Total of Knowledge of HPV in females	1.82 ± 1.73				
	Low	205 (66.8%)				
	Moderate	36 (11.7%)				
	High	66 (21.5%)				
	Total of Knowledge of HPV	4.59 ± 3.06				
	Low	224 (73.0%)				
	Moderate	81 (26.4%)				
	High	2 (0.7%)				

**Table 3:** Levels for attitude toward HPV vaccination. (n = 307).

	Attitude Toward HPV Vaccination	Mean ± SD	Low		Moderate		High	
			No	%	No	%	No	%
1	We should vaccinate males against HPV in order to protect their future partners from cervical cancer and other	3.96 ± 1.12	25	8.1	88	28.7	194	63.2
2	We already vaccinate females against HPV so there is no need to vaccinate males as well.	2.84 ± 1.42	131	42.7	85	27.7	91	29.6
3	It would be important to vaccinate females against HPV to prevent them from getting genital and anal warts	4.11 ± 0.92	8	2.6	86	28.0	213	69.4
4	HPV causes too few cancers among females to make it worthwhile to vaccinate them	3.64 ± 1.02	25	8.1	141	45.9	141	45.9
5	Vaccinating females does not make sense because genital and anal warts can be managed in other ways	2.97 ± 1.32	110	35.8	109	35.5	88	28.7
6	It would be important to vaccinate females against HPV to prevent males from getting infected with HPV	3.51 ± 1.17	45	14.7	124	40.4	138	45.0
7	It is too late to vaccinate against HPV if an adolescent female is already sexually active	2.83 ± 1.35	118	38.4	108	35.2	81	26.4
	Total of attitude toward female vaccination	23.86 ± 5.52	70	22.8	171	55.7	66	21.5
1	I feel that the vaccine is too new and “hasn’t been around long enough”.	3.64 ± 1.16	46	15.0	91	29.6	170	55.4
2	I am concerned about the safety of HPV vaccine	3.44 ± 1.28	78	25.4	78	25.4	151	49.2
3	I am concerned about the efficacy of HPV vaccine	3.40 ± 1.26	79	25.7	78	25.4	150	48.9
4	I am opposed to HPV vaccination for moral or religious reasons	2.57 ± 1.45	159	51.8	76	24.8	72	23.5
5	I am unaware that the vaccine is available for both males and females	3.69 ± 1.24	48	15.6	86	28.0	173	56.4
6	I am interested in the HPV vaccine for females	3.92 ± 1.12	34	11.1	72	23.5	201	65.5
7	I am more comfortable providing the HPV vaccine to females than males	3.34 ± 1.30	77	25.1	96	31.3	134	43.7
8	I am concerned about the cost of the vaccine	3.31 ± 1.32	76	24.8	98	31.9	133	43.3
	Total of attitude toward vaccine	27.33 ± 6.71	76	24.8	158	51.5	73	23.8
	Total of Attitude toward HPV vaccination	51.18 ± 11.16	77	25.1	175	57.0	55	17.9

**Table 4:** Correlation matrix between the study variables and demographics (n = 307).

		Age	Marital Statues	GPA	Year	Family History of Cervical Cancer	Receive HPV Vaccine	Knowledge of HPV in General	HPV Knowledge in Females	Overall, Knowledge	Attitudes toward HPV Vaccination in Females	Attitudes toward the HPV Vaccine in General	Overall Attitude
Age	r												
	*p*												
Marital statues	r	−0.048											
	*p*	0.403											
GPA	r	−0.039	0.050										
	*p*	0.499	0.387										
Year	r	0.745 *	0.082	0.009									
	*p*	<0.001 *	0.153	0.873									
Family history of cervical cancer	r	−0.114 *	0.134 *	0.197 *	−0.204 *								
	*p*	0.046 *	0.019 *	0.001 *	<0.001 *								
Receive HPV vaccine	r	−0.021	0.314 *	0.161 *	−0.061	0.267 *							
	*p*	0.710	<0.001 *	0.005 *	0.290	<0.001 *							
Knowledge of HPV in general	r	−0.141 *	0.088	0.227 *	−0.143 *	0.281 *	0.169 *						
	*p*	0.014 *	0.126	<0.001 *	0.012 *	<0.001 *	0.003 *						
HPV knowledge in females	r	−0.127 *	0.175 *	0.256 *	−0.089	0.397 *	0.207 *	0.518 *					
	*p*	0.026 *	0.002 *	<0.001 *	0.122	<0.001 *	<0.001 *	<0.001 *					
Overall Knowledge	r	−0.154 *	0.150 *	0.277 *	−0.133 *	0.388 *	0.215 *	0.875 *	0.867 *				
	*p*	0.007 *	0.009 *	<0.001 *	0.019 *	<0.001 *	<0.001 *	<0.001 *	<0.001 *				
Attitudes toward HPV vaccination in females	r	−0.223 *	0.162 *	0.251 *	−0.288 *	0.391 *	0.196 *	0.420 *	0.592 *	0.579 *			
	*p*	<0.001 *	0.004 *	<0.001 *	<0.001 *	<0.001 *	0.001 *	<0.001 *	<0.001 *	<0.001 *			
Attitudes toward the HPV vaccine in general	r	−0.318 *	0.193 *	0.200 *	−0.364 *	0.372 *	0.164 *	0.361 *	0.481 *	0.483 *	0.663 *		
	*p*	<0.001 *	0.001 *	<0.001 *	<0.001 *	<0.001 *	0.004 *	<0.001 *	<0.001 *	<0.001 *	<0.001 *		
Overall Attitude	r	−0.301 *	0.196 *	0.244 *	−0.361 *	0.417 *	0.195 *	0.425 *	0.582 *	0.577 *	0.893 *	0.929 *	
	*p*	<0.001 *	0.001 *	<0.001 *	<0.001 *	<0.001 *	0.001 *	<0.001 *	<0.001 *	<0.001 *	<0.001 *	<0.001 *	

r: Pearson coefficient, *: Statistically significant at *p* ≤ 0.05.

**Table 5:** Multiple Linear Regression Analysis Showing the effect of demographics on knowledge and attitude (n = 307).

Demographics	Knowledge						Attitude					
	B	Beta	*t*	*p*	95% CI		B	Beta	*t*	*p*	95% CI	
					LL	UL					LL	UL
Age	−5.358	−0.116	1.478	0.141	−12.495	1.778	0.413	0.010	0.161	0.872	−4.641	5.466
Marital status	8.734	0.068	1.223	0.222	-5.323	22.790	14.814	0.135	2.932 *	0.004 *	4.872	24.756
GPA	6.318	0.196	3.708 *	<0.001 *	2.965	9.671	2.515	0.091	2.046 *	0.042 *	0.096	4.934
Year	0.278	0.013	0.164	0.869	−3.050	3.606	−5.235	−0.291	4.387 *	<0.001 *	−7.583	−2.887
Family history of cervical cancer	12.244	0.309	5.596 *	<0.001 *	7.938	16.550	5.349	0.158	3.297 *	0.001 *	2.157	8.542
Receive HPV vaccine	5.519	0.078	1.400	0.163	−2.238	13.277	−0.898	−0.015	−0.322	0.748	−6.389	4.593
Knowledge	-	-	-	-	-	-	0.371	0.436	9.113 *	<0.001 *	0.291	0.451
R^2^ = 0.217, F = 13.860 *, *p* < 0.001 *							R^2^ = 0.465, F = 37.146 *, *p* < 0.001 *					

F, *p*: f and *p* values for the model. R^2:^ Coefficient of determination. B: Unstandardized Coefficients. Beta: Standardized Coefficients. *t*: *t*-test of significance. LL: Lower limit. UL: Upper Limit. *: Statistically significant at *p* ≤ 0.05.

## Data Availability

The datasets generated and/or analyzed during the current study are not publicly available due to data privacy but are available from the corresponding author on reasonable request.

## Supplementary Materials

The following supporting information can be downloaded at: https://www.mdpi.com/article/10.3390/healthcare11121766/s1, Supplementary Table S1: Total population and study sample size; Supplementary Table S2: sampling calculation (95% Confidence, 5% error).

## References

[1] Das R., Mishra V., Sharma N., Khurana N. (2018). Human papillomavirus and its nature of infection: An overview. Asian J. Pharm. Clin. Res..

[2] Chin-Hong P.V., Reid G.E., AST Infectious Diseases Community of Practice (2019). Human papillomavirus infection in solid organ transplant recipients: Guidelines from the American Society of Transplantation Infectious Diseases Community of Practice. Clin. Transplant..

[3] Warts: HPV, Causes, Types, Treatments, Removal, Prevention. Cleveland Clinic. https://my.clevelandclinic.org/health/diseases/15045-warts.

[4] Kombe A.J.K., Li B., Zahid A., Mengist H.M., Bounda G.-A., Zhou Y., Jin T. (2021). Epidemiology and Burden of Human Papillomavirus and Related Diseases, Molecular Pathogenesis, and Vaccine Evaluation. Front. Public Health.

[5] National Cancer Institute Parent Concerns about HPV Vaccine Safety Increasing. 15 November 2022, 2 November 2022, & 6 October 2022. https://www.cancer.gov/news-events/cancer-currents-blog/2021/hpv-vaccine-parents-safety-concerns.

[6] Tepe N.B., Ozcorekci O. (2020). Knowledge about the human papillomavirus among high school and university students a comprehensive questionnaire study from Southeast Turkey. J. Obstet. Gynaecol. Res..

[7] Bodson J., Wilson A., Warner E.L., Kepka D. (2017). Religion and HPV vaccine-related awareness, knowledge, and receipt among insured women aged 18–26 in Utah. PLoS ONE.

[8] Bal-Yılmaz H., Koniak-Griffin D. (2017). Knowledge, Behaviors, and Attitudes About Human Papilloma Virus Among Nursing Students in Izmir, Turkey. J. Cancer Educ..

[9] White L., Waldrop J., Waldrop C. (2016). Human Papillomavirus and Vaccination of Males: Knowledge and Attitudes of Registered Nurses. Pediatr. Nurs..

[10] Lin W., Wang Y., Liu Z., Chen B., Yuan S., Wu B., Gong L. (2019). Awareness and attitude towards human papillomavirus and its vaccine among females with and without daughter(s) who participated in cervical cancer screening in Shenzhen, China. Trop. Med. Int. Health.

[11] Almaghlouth A.K., Bohamad A.H., Alabbad R.Y., Alghanim J.H., Alqattan D.J., Alkhalaf R.A. (2022). Acceptance, Awareness, and Knowledge of Human Papillomavirus Vaccine in Eastern Province, Saudi Arabia. Cureus.

[12] Alqarawi S.A., Aljarbooa E.F., Almuqaytib A.Y., Alomar I.A., Altwaijri M.H., Aldakhil A.Y., Altowaijri A.H. (2023). Assessment of saudi females’ knowledge regarding human papillomavirus infection, screening, and available methods for prevention: A cross-sectional study in qassim region. Cureus.

[13] (2022). Centers for Disease Control and Prevention. Std Facts—Human Papillomavirus (HPV). Centers for Disease Control and Prevention. https://www.cdc.gov/std/hpv/stdfact-hpv.htm.

[14] Darraj A.I., Arishy A.M., Alshamakhi A.H., Osaysi N.A., Jaafari S.M., Sumayli S.A., Mushari R.Y., Alhazmi A.H. (2022). Human Papillomavirus Knowledge and Vaccine Acceptability in Jazan Province, Saudi Arabia. Vaccines.

[15] Berenson A.B., Hirth J.M., Chang M., Kuo Y.-F., Richard P., Jones D.L. (2021). A brief educational intervention can improve nursing students’ knowledge of the human papillomavirus vaccine and readiness to counsel. Hum. Vaccines Immunother..

[16] Taebi M., Riazi H., Keshavarz Z., Afrakhteh M. (2019). Knowledge and Attitude Toward Human Papillomavirus and HPV Vaccination in Iranian Population: A Systematic Review. Asian Pac. J. Cancer Prev..

[17] Burd E.M. (2003). Human papillomavirus and cervical cancer. Clin. Microbiol. Rev..

[18] (2021). Facts About HPV for Adults. National Foundation for Infectious Diseases. https://www.nfid.org/infectious-diseases/facts-about-human-papillomavirus-hpv-for-adults/.

[19] Cinar I.O., Ozkan S., Aslan G.K., Alatas E. (2019). Knowledge and Behavior of University Students toward Human Papillomavirus and Vaccination. Asia-Pac. J. Oncol. Nurs..

[20] McBride K.R., Singh S. (2017). Predictors of Adults’ Knowledge and Awareness of HPV, HPV-Associated Cancers, and the HPV Vaccine: Implications for Health Education. Health Educ. Behav..

[21] Chen G., Wu B., Dai X., Zhang M., Liu Y., Huang H., Mei K., Wu Z. (2021). Gender Differences in Knowledge and Attitude towards HPV and HPV Vaccine among College Students in Wenzhou, China. Vaccines.

[22] Monteiro D.L.M., Brollo L.C.S., De Souza T.P., Dos Santos J.R.P., Santos G.R., Correa T., Da Costa J.T., De Oliveira M.A.P., Trajano A.J.B. (2018). Knowledge on the HPV Vaccine among University Students. Rev. Inst. Med. Trop. São Paulo.

[23] Patel H., Jeve Y.B., Sherman S.M., Moss E.L. (2016). Knowledge of human papillomavirus and the human papillomavirus vaccine in European adolescents: A systematic review. Sex. Transm. Infect..

[24] Khatiwada M., Kartasasmita C., Mediani H.S., Delprat C., Van Hal G., Dochez C. (2021). Knowledge, Attitude and Acceptability of the Human Papilloma Virus Vaccine and Vaccination among University Students in Indonesia. Front. Public Health.

[25] Inguva S., Barnard M., Ward L.M., Yang Y., Pittman E., Banahan B.F., Kirby T.R., Noble S.L. (2020). Factors influencing Human papillomavirus (HPV) vaccination series completion in Mississippi Medicaid. Vaccine.

[26] Zdemir S., Akkaya R., Karaşahin K. (2020). Analysis of community-based studies related with knowledge, aware-ness, attitude, and behaviors towards HPV and HPV vaccine published in Turkey: A systematic review. J. Turk. Ger. Gynecol. Assoc..

[27] Dönmez S., Öztürk R., Kısa S., Weller B.K., Zeyneloğlu S. (2018). Knowledge and perception of female nursing students about human papillomavirus (HPV), cervical cancer, and attitudes toward HPV vaccination. J. Am. Coll. Health.

[28] Montesdeoca A., Sánchez A., Vergés E., López N. (2022). Factors influencing HPV knowledge and vaccine acceptability in parents of adolescent children: Results from a survey-based study (KAPPAS study). Hum. Vaccines Immunother..

[29] Altamimi T. (2020). Human papillomavirus and its vaccination Knowledge and attitudes among female university students in Saudi Arabia. J. Fam. Med. Prim. Care.

[30] Kisaakye E., Namakula J., Kihembo C., Kisakye A., Nsubuga P., Babirye J.N. (2018). Level and factors associated with uptake of human papillomavirus infection vaccine among female adolescents in Lira District, Uganda. Pan Afr. Med. J..

[31] Pelullo C.P., Esposito M.R., Di Giuseppe G. (2019). Human papillomavirus infection and vaccination: Knowledge and attitudes among nursing students in Italy. Int. J. Environ. Res. Public Health.

[32] He J., He L. (2018). Knowledge of HPV and acceptability of HPV vaccine among women in western China: A cross-sectional survey. BMC Women’s Health.

[33] Sallam M., Al-Mahzoum K., Eid H., Assaf A.M., Abdaljaleel M., Al-Abbadi M., Mahafzah A. (2021). Attitude towards HPV Vaccination and the Intention to Get Vaccinated among Female University Students in Health Schools in Jordan. Vaccines.

[34] Shakurnia A., Ghadiri A., Hamidi M., Jelodar N. (2022). Knowledge and Attitude of Midwifery Students toward Human Papilloma Virus Infection and Cervical Cancer at Ahvaz Jundishapur University of Medical Sciences, Iran. J. Res. Dev. Nurs. Midwifery.

[35] Shetty S., Prabhu S., Shetty V., Shetty A.K. (2019). Knowledge, attitudes and factors associated with acceptability of human papillomavirus vaccination among undergraduate medical, dental and nursing students in South India. Hum. Vaccines Immunother..

[36] Ebu N.I., Abotsi-Foli G.E., Gakpo D.F. (2021). Nurses’ and midwives’ knowledge, attitudes, and acceptance regarding human papillomavirus vaccination in Ghana: A cross-sectional study. BMC Nurs..

[37] Shakurnia A., Ghadiri A., Jelodar N., Hamidi M. (2022). Awareness and Knowledge of Medical, Nursing and Midwifery Students About Human Papillomavirus Infection and its Vaccine in Ahvaz Jundishapur University of Medical Sciences in 2020. Prev. Care Nurs. Midwifery J..

[38] Farsi N.J., Baharoon A.H., Jiffri A.E., Marzouki H.Z., Merdad M.A., Merdad L.A. (2021). Human papillomavirus knowledge and vaccine acceptability among male medical students in Saudi Arabia. Hum. Vaccines Immunother..

[39] Kellogg C., Shu J., Arroyo A., Dinh N.T., Wade N., Sanchez E., Equils O. (2019). A significant portion of college students are not aware of HPV disease and HPV vaccine recommendations. Hum. Vaccines Immunother..

[40] Yörük S., Açıkgöz A., Ergör G. (2016). Determination of knowledge levels, attitude and behaviors of female university students concerning cervical cancer, human papiloma virus and its vaccine. BMC Women’s Health.

[41] Oh K.M., Alqahtani N., Chang S., Cox C. (2021). Knowledge, beliefs, and practice regarding human papillomavirus (HPV) vaccination among American college students: Application of the health belief model. J. Am. Coll. Health.

[42] Singh J., Baliga S.S. (2021). Knowledge regarding cervical cancer and HPV vaccine among medical students: A cross-sectional study. Clin. Epidemiol. Glob. Health.

[43] Hollins A., Wardell D., Fernandez M.E., Markham C., Guilamo-Ramos V., Maria D.S. (2021). Human Papillomavirus Vaccination Status and Parental Endorsement Intentions among Undergraduate Student Nurses. Int. J. Environ. Res. Public Health.

[44] D’Errico M.P.D., Tung W.-C.P., Lu M.P., D’Errico R.D. (2020). Knowledge, attitudes, and practices related to human papillomavirus vaccination among college students in a state university: Implications for nurse practitioners. J. Am. Assoc. Nurse Pract..

