# Supplementary material for: Knowledge and Attitudes toward Human Papillomavirus and Vaccination: A Survey among Nursing Students in Saudi Arabia
Source: Healthcare (Basel). 2023 Jun 15;11(12):1766. doi: 10.3390/healthcare11121766 (PMC10297885; doi:10.3390/healthcare11121766)
Supplement: Supplementary file 1 [file healthcare-11-01766-s001.zip › healthcare-2428651-supplementary.pdf]

List of supplementary tables

Supplementary Table S1: Total population and study sample size.

College	No.student	Formula
Nursing	1014	307

Supplementary Table S2: sampling calculation (95% Confidence, 5% error)

Year	Total population	Calculated population
1st year	517	156
2nd year	250	77
3rd year	129	39
4th year	118	35
